# Unlocking translational resilience for mRNA vaccines by viral UTRs

**DOI:** 10.1016/j.omtn.2026.102969

**Published:** 2026-06-09

**Authors:** Jordan J. Minnell, Jochem N.A. Vink, Rebecca E. McKenzie

**Affiliations:** 1 Malaghan Institute of Medical Research, Wellington, New Zealand; 2 Ferrier Research Institute, Victoria University of Wellington, Lower Hutt, New Zealand

mRNA vaccines have revolutionized infectious disease prevention, yet they face a fundamental limitation: their exclusive reliance upon cap-dependent translation to express antigen. This mechanism of translation can become severely compromised under physiological stress conditions that are commonly induced at vaccination sites. In this issue of *Molecular Therapy Nucleic Acids*, Seo et al.^1^ present a solution by demonstrating that a SARS-CoV-2-derived internal ribosome entry site (IRES) element can enable continued protein expression under hypoxia and inflammation (Figure 1). Remarkably, lipid nanoparticle (LNP) vaccines containing this viral element, but synthesized without expensive nucleotide modifications, generated immune responses equal to or exceeding those of current clinical vaccines. These findings challenge the current paradigm that requires expensive nucleotide modifications for effective mRNA vaccines. More broadly, it provides an example of how viral translation strategies could unlock new therapeutic potential for mRNA platforms.

**Figure 1:**
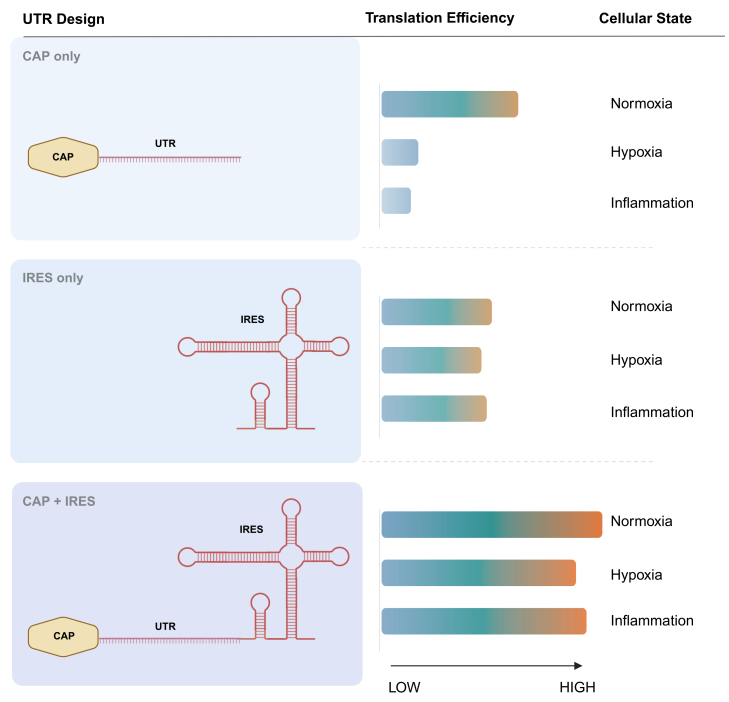
Depiction of cap-mediated, IRES-mediated and hybrid UTR-mediated protein translation efficiency under different cellular stresses Created in BioRender. Mckenzie, R. (2026) https://BioRender.com/cw3x5o7.

Current clinical mRNA vaccines rely on several key features to drive protein expression. Both a 5′-cap and 3′-polyA tail are necessary for ribosomal translation, while incorporation of nucleotides such as N1-methylpseudouridine (m^1^Ψ-U) or pseudouridine (Ψ-U) help evade pattern recognition receptors (PRRs).^2^^,^^3^ However, these principles assume a permissive translational environment, which is not always guaranteed. mRNA therapies must function in physiological conditions where translation is actively suppressed, including in cells exposed to type I interferons or undergoing metabolic stress: cellular states associated with LNP transfection.^4^ Hypoxic conditions, which commonly occur in poorly vascularized tissues like the tumor microenvironment or during intense immune activation, can further compromise translation through mammalian target of rapamycin (mTOR) pathway suppression and eukaryotic translation initiation factor 4E (eIF4E) sequestration.^5^

The Nobel Prize-winning work^2^ established a field consensus that nucleotide modification is essential for therapeutic mRNA to mitigate the reactogenicity driven by uridines immunostimulatory interactions with PRRs. However, systematic analysis has since revealed that manufacturing process optimization can reduce (but not eliminate) unmodified mRNA immunostimulation.^6^ Despite current advances in mRNA manufacturing techniques (including engineered T7 polymerase variants and RNA chromatography methods), the therapeutic potential of uridine-based systems remains underexplored. Translational restriction during vaccination is not solely a nucleotide problem, as the LNP itself triggers the innate immune system during transfection regardless of whether it contains m^1^Ψ-U.^4^ Viruses have evolved under precisely these conditions and are forced to adopt translational strategies that can favor viral replication over host protein synthesis,^1^^,^^7^^,^^8^ providing a rich source of blueprints for the next wave of RNA therapeutics.

Seo et al. used a SARS-CoV-2 subgenomic replicon system to reveal that while viral expression of structural genes diminished by 90% under hypoxic conditions, non-structural protein translation remained stable. This led to identification of IRES activity in a stem-loop structure (SL4.5-5) present in genomic but not subgenomic viral RNA. Surprisingly, comparative analysis across SARS-CoV-2 variants revealed that the ancestral Wuhan strain possessed superior IRES activity compared to later variants like Delta, due to mutations in a conserved 5′-UUUCGU-3′ motif within the SL5 loops that proved essential for function. When incorporated downstream of clinical vaccine 5′ UTR sequences from the Pfizer and Moderna platforms, SL4.5-5 created hybrid constructs that enabled both cap-dependent and cap-independent translation, consistently outperforming either UTR alone across multiple cell lines, with three different spike protein variants. This dual translation mechanism provided superior performance by combining the efficiency of conventional cap-dependent initiation with the stress-resistant backup of IRES-mediated translation.^8^

The mechanistic advantages translated into remarkable vaccine performance *in vivo*. When encapsulated and delivered via LNPs, vaccines with the hybrid UTR elicited strong humoral and cellular adaptive immune responses while retaining low inflammatory profiles at therapeutic doses, in mice. Critically, direct comparison of unmodified versus pseudouridine-modified vaccines revealed similar cytokine responses, with both showing dose-dependent increases in IL-6, IFN-α, and IFN-γ only at high doses (10 μg), suggesting the enhanced performance stemmed from improved translation efficiency rather than PRR evasion. Most impressively, unmodified mRNA vaccines harboring the hybrid UTR achieved 2.7-fold higher anti-RBD antibody titers than Pfizer’s modified Raxtozinameran when tested head-to-head against the same Omicron XBB.1.5 variant in a challenge model. The vaccines also elicited robust T cell responses (∼2,000 IFN-γ spots per million splenocytes) and provided complete protection against viral challenge, with no detectable infectious virus particles in the lungs. These results demonstrate that strategic UTR design can achieve clinical-grade performance without expensive nucleotide modifications.

The opportunities enabled by this novel design are far-reaching. The ability to achieve expression across a broader range of cellular metabolic states could allow RNA therapeutics to penetrate and function more effectively within hypoxic tumors or infected cells. In addition, a major limitation of current RNA vaccines is the need to balance efficient payload translation with immune activation, as these processes often compete with one another.^9^ By decoupling immune activation from antigen expression through the removal of the strict dependence on cap-dependent translation, vaccines could be engineered to elicit stronger immune responses without compromising protein production. Incorporating IRES elements into linear mRNA may also create opportunities for the development of novel cap analogs. Existing cap analogs are constrained by the requirement for recognition by the cellular translation machinery, whereas reduced reliance on cap-dependent initiation could permit alternative cap chemistries optimized for enhanced RNA stability or localization. Finally, the prospect of producing functional vaccines using unmodified nucleotides could reduce manufacturing costs, reduce the risk of aberrant frameshift protein production associated with nucleotide modifications,^10^ and democratize RNA therapeutics by moving away from licensed technologies.

Despite these promising opportunities, significant challenges remain before IRES-driven linear mRNA platforms can achieve widespread clinical adoption. The activity of IRES elements is often highly context-dependent, varying across cell types, physiological conditions, and transcript architectures, which may complicate predictable therapeutic design. By challenging the assumption that efficient mRNA therapeutics must rely exclusively on canonical cap-dependent translation, this emerging approach expands the conceptual design space of RNA medicines. As the field continues to mature, IRES-enabled mRNA systems may represent an important step toward more versatile, accessible, and physiologically resilient RNA therapeutics.

## Acknowledgments

J.J.M., J.N.A.V., and R.E.M. were supported by a New Zealand Ministry of Business Innovation and Employment (MBIE) Strategic Scientific Investment Fund (MA-006990). J.J.M. was also supported by an MBIE Independent Research Organisation Fund (MALINSMEDRES2302).

## Author contributions

Writing, J.J.M., J.N.A.V., and R.E.M.

## Declaration of interests

The authors declare no competing interests.
